# Supplementary material for: Long-term, age-associated activity quantification in the DE50-MD dog model of Duchenne muscular dystrophy
Source: Dis Model Mech. 2025 Jul 14;18(7):dmm052135. doi: 10.1242/dmm.052135 (PMC12309892; doi:10.1242/dmm.052135)
Supplement: Supplementary information [file dmm-18-052135-s1.pdf]

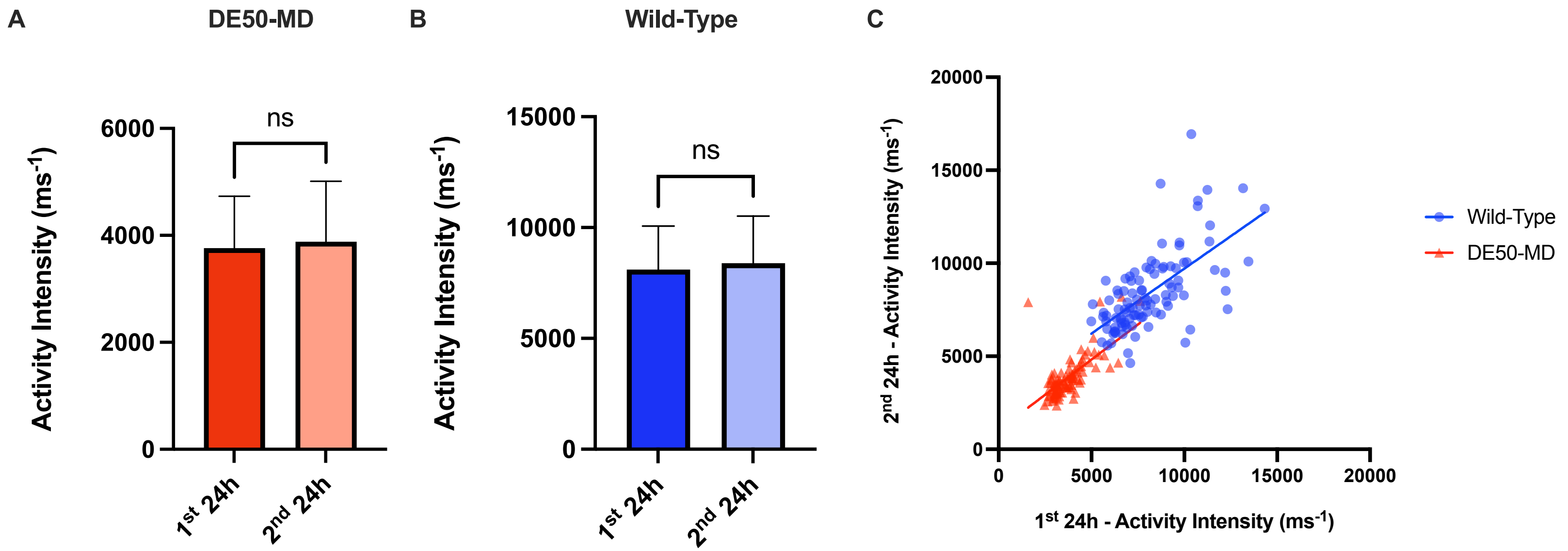

Fig. S1. Activity Intensity (mean and standard deviation) metrics computed for the 1st and 2nd 24h periods for (A) DE50-MD dogs (red; *n*=11 dogs; *n*=95 sets of 48h activity monitoring recordings in total), (B) wild-type dogs (blue; *n*=14 dogs; *n*=103 sets of 48h activity monitoring recordings in total) and (C) the correlation between the results for the two time periods. Note the lack of any significant difference in Activity Intensity (ns; $P>0.05$) in the second period compared with the first in either genotype. Recordings within each animal between first and second 24 hour period were highly correlated for each genotype ($P<0.0001$; WT: slope estimate: 0.75 +/- 0.09 SE; DE50-MD: slope estimate: 0.69 +/- 0.08 SE).

Disease Models & Mechanisms • Supplementary information

Table S1. Activity metrics involving time spent above or below an acceleration threshold for DE50-MD and WT dogs, and *n* numbers at all ages.

Age in months	*n*		% time spent at rest***		% time spent at low intensity***		% time high intensity**		Average bout duration low intensity [s]*		Total bout count low intensity*		Average bout duration high intensity [s]***		Total bout count high intensity***	
	DE50	WT	DE50	WT	DE50	WT	DE50	WT	DE50	WT	DE50	WT	DE50	WT	DE50	WT
**3**	3	13	80.5 (2.9)	74.5 (4.5)	18.9 (2.8)	23.6 (4.5)	0.7 (0.2)	2.0 (0.6)	2.1 (0.4)	2.5 (0.6)	8175[f] (1802)	9005[f] (1306)	0.6 (0.5)	0.8 (0.1)	995 (147)	2118 (580)
**4**	5	13	83.4 (2.7)	75.5 (4.0)	15.9 (2.5)	22.2 (3.4)	0.7 (0.3)	2.3 (1.0)	2.1 (0.5)	2.7 (0.7)	6977 (1649)	8081 (1471)	0.7 (0.1)	0.9 (0.1)	856 (369)	2196 (646)
**5**	4	8	86.2 (2.5)	78.4 (3.2)	13.5 (2.5)	19.9 (3.2)	0.3 (0.1)	1.7 (0.8)	1.8 (0.4)	2.2 (0.5)	6774 (1369)	8722 (972)	0.7 (0.0)	0.8 (0.1)	366 (134)	1821 (671)
**6**	6	7	88.5 (1.2)	79.9 (3.1)	11.2 (1.1)	18.5 (2.6)	0.3 (0.1)	1.6 (0.5)	1.6 (0.2)	2.2 (0.5)	6157 (543)	7894 (780)	0.6 (0.0)	0.8 (0.1)	407 (136)	1787 (472)
**7**	3	6	89.4 (1.5)	82.4 (2.0)	10.4 (1.4)	16.3 (2.1)	0.3 (0.1)	1.4 (0.2)	1.6 (0.2)	2.1 (0.2)	5757 (736)	7220 (966)	0.7 (0.1)	0.8 (0.1)	319 (124)	1558 (214)
**8**	7	6	89.9 (2.0)	84.1 (1.3)	9.8 (1.9)	14.7 (0.9)	0.3 (0.1)	1.2 (0.5)	1.5 (0.3)	2.0 (0.1)	5710 (480)	7015 (705)	0.8 (0.1)	0.8 (0.1)	330 (95)	1285 (332)
**9**	7	6	90.7 (2.1)	82.4 (1.7)	9.1 (2.0)	16.3 (1.7)	0.2 (0.1)	1.4 (0.3)	1.5 (0.2)	2.2 (0.3)	5477 (474)	7146 (556)	0.7 (0.1)	0.8 (0.1)	278 (164)	1524 (236)
**10**	8	8	90.8 (2.5)	82.1 (2.2)	8.9 (2.4)	16.2 (1.8)	0.3 (0.1)	1.7 (0.9)	1.6 (0.2)	2.3 (0.4)	5088 (1227)	6827 (975)	0.7 (0.1)	0.8 (0.2)	307 (146)	1763 (497)
**11**	8	8	92.0 (1.2)	81.3 (2.6)	7.8 (1.1)	17.0 (2.2)	0.2 (0.1)	1.6 (0.7)	1.4 (0.1)	2.3 (0.4)	4933 (715)	7215 (1013)	0.8 (0.1)	0.8 (0.2)	229 (75)	1618 (389)
**12**	6	8	92.4 (1.7)	81.1 (3.4)	7.4 (1.6)	17.0 (2.6)	0.2 (0.1)	1.9 (1.2)	1.4 (0.1)	2.4 (0.6)	4599 (669)	6885 (922)	0.8 (0.1)	0.9 (0.2)	215 (87)	1785 (657)
**13**	7	8	90.9 (1.7)	82.8 (3.0)	8.8 (1.5)	15.6 (2.4)	0.3 (0.1)	1.6 (0.7)	1.4 (0.1)	2.4 (0.5)	5485[f] (689)	6323[f] (1028)	0.8 (0.1)	0.8 (0.1)	305 (144)	1590 (509)
**14**	7	4	92.7 (0.7)	80.8 (5.5)	7.1 (0.7)	16.2 (4.0)	0.2 (0.1)	3.0 (1.7)	1.4 (0.1)	2.7 (0.8)	4619 (453)	6173 (904)	0.8 (0.2)	1.1 (0.2)	223 (75)	2220 (955)
**15**	7	3	92.6 (1.3)	83.3 (2.8)	7.3 (1.2)	14.5 (1.2)	0.2 (0.1)	2.2 (0.4)	1.4 (0.1)	2.4 (0.3)	4707 (832)	6049 (760)	0.7 (0.1)	1.1 (0.1)	225 (84)	1784 (287)
**16**	8	3	93.2 (1.2)	82.8 (3.8)	6.7 (1.2)	14.9 (2.4)	0.2 (0.1)	2.4 (1.6)	1.4 (0.1)	2.5 (0.7)	4390 (741)	6122 (414)	0.8 (0.1)	1.0 (0.2)	226 (85)	1923 (993)
**17**	8	3	93.0 (0.9)	82.2 (1.8)	6.7 (0.9)	14.9 (1.7)	0.2 (0.1)	2.9 (0.5)	1.3 (0.1)	2.6 (0.4)	4561 (565)	5944 (755)	0.9 (0.2)	1.2 (0.1)	206 (61)	2061 (284)
**18**	8	3	93.3 (0.8)	84.9 (0.4)	6.5 (0.8)	13.5 (0.5)	0.2 (0.0)	1.6 (0.2)	1.4 (0.1)	2.2 (0.2)	4310 (432)	6009 (395)	0.8 (0.2)	0.9 (0.2)	164 (36)	1513 (120)

Results for metrics from left to right: % time spent at rest, % time spent at low intensity activity, average active bout duration at low intensity (s), total active bout count at low intensity activity, average bout duration at high intensity activity (s), total bout count at high intensity activity for DE50-MD and WT control dogs. Mean (standard deviation) displayed for each metric at each age between 3 and 18 months. Metrics counting discrete bouts have been rounded to the nearest integer, acceleration metrics (MX_{ACC}) to two decimal places, and all other metrics to one decimal place. Symbols indicate group effect at all ages unless noted otherwise: * P<0.05, ** P<0.01, *** P<0.001; ^{f} not significant at that age point

Table S2. Activity intensity, MX_{ACC}, PC1 and PC2 for DE50-MD and WT dogs at all ages.

Age in months	Activity Intensity [ms^{-1}]***		$M2_{ACC}$ [*g*]**		$M30_{ACC}$ [*g*]**		$M60_{ACC}$ [*g*]**		PC1***		PC2	
	DE50	WT	DE50	WT	DE50	WT	DE50	WT	DE50	WT	DE50	WT
**3**	7101 (894)	9872 (1878)	1.14 (0.11)	1.63 (0.15)	0.52 (0.02)	0.72 (0.10)	0.39 (0.02)	0.53 (0.08)	-0.5 (1.0)	-3.6 (1.8)	2.5 (0.5)	2.0 (0.7)
**4**	6151 (1082)	10068 (1950)	1.09 (0.12)	1.7 (0.19)	0.48 (0.10)	0.79 (0.16)	0.35 (0.08)	0.56 (0.11)	0.3 (1.2)	-3.9 (1.9)	1.5 (0.6)	1.2 (1.0)
**5**	5088 (807)	8759 (1718)	0.96 (0.14)	1.75 (0.36)	0.38 (0.05)	0.68 (0.14)	0.29 (0.03)	0.48 (0.10)	1.4 (0.8)	-2.5 (1.8)	1.2 (0.6)	1.5 (0.7)
**6**	4551 (382)	8235 (1270)	0.95 (0.08)	1.65 (0.18)	0.37 (0.04)	0.67 (0.08)	0.28 (0.03)	0.47 (0.07)	2.1 (0.5)	-2.1 (1.3)	1.0 (0.3)	1.2 (0.6)
**7**	4251 (484)	7303 (560)	0.93 (0.15)	1.63 (0.10)	0.34 (0.03)	0.63 (0.05)	0.25 (0.02)	0.44 (0.04)	2.3 (0.6)	-1.3 (0.5)	0.6 (0.3)	0.9 (0.7)
**8**	4225 (456)	6591 (694)	0.99 (0.14)	1.53 (0.16)	0.33 (0.05)	0.57 (0.10)	0.25 (0.03)	0.39 (0.06)	2.3 (0.8)	-0.5 (1.0)	0.1 (0.5)	0.7 (0.7)
**9**	3823 (673)	7320 (615)	0.83 (0.14)	1.66 (0.20)	0.31 (0.05)	0.63 (0.06)	0.24 (0.04)	0.43 (0.05)	2.9 (0.8)	-1.2 (0.7)	0.5 (0.4)	0.7 (0.8)
**10**	3825 (773)	7735 (1325)	0.91 (0.19)	1.78 (0.34)	0.33 (0.05)	0.68 (0.15)	0.24 (0.04)	0.48 (0.11)	2.7 (0.9)	-1.8 (1.8)	0.1 (0.7)	0.3 (1.3)
**11**	3571 (322)	7825 (1296)	0.87 (0.13)	1.74 (0.30)	0.29 (0.03)	0.67 (0.13)	0.22 (0.02)	0.46 (0.09)	3.0 (0.4)	-1.9 (1.6)	-0.3 (0.6)	0.5 (1.1)
**12**	3358 (481)	8262 (2124)	0.86 (0.19)	1.94 (0.57)	0.28 (0.02)	0.71 (0.19)	0.21 (0.02)	0.49 (0.14)	3.1 (0.7)	-2.2 (2.4)	-0.5 (0.6)	0.1 (1.3)
**13**	3886 (607)	7416 (1545)	0.96 (0.20)	1.77 (0.26)	0.32 (0.05)	0.66 (0.14)	0.24 (0.03)	0.44 (0.10)	2.7 (0.8)	-1.5 (1.8)	0.2 (0.4)	0.0 (0.8)
**14**	3257 (244)	9229 (3026)	0.86 (0.11)	2.09 (0.32)	0.29 (0.02)	0.88 (0.24)	0.21 (0.01)	0.6 (0.20)	3.3 (0.3)	-3.9 (3.5)	-0.3 (0.8)	-1.6 (1.3)
**15**	3327 (455)	7744 (926)	0.83 (0.10)	2.02 (0.28)	0.29 (0.03)	0.77 (0.08)	0.21 (0.02)	0.48 (0.06)	3.3 (0.4)	-2.8 (1.0)	-0.2 (0.6)	-1.5 (1.3)
**16**	3184 (410)	8278 (2617)	0.86 (0.13)	2.16 (0.69)	0.28 (0.02)	0.76 (0.29)	0.2 (0.02)	0.53 (0.21)	3.4 (0.5)	-2.4 (2.8)	-0.4 (0.6)	-0.9 (1.3)
**17**	3220 (283)	8671 (877)	0.88 (0.11)	2.14 (0.08)	0.28 (0.02)	0.9 (0.09)	0.2 (0.01)	0.57 (0.08)	3.3 (0.3)	-3.5 (1.5)	-0.7 (0.7)	-1.7 (0.6)
**18**	3081 (264)	6948 (156)	0.8 (0.06)	1.9 (0.07)	0.27 (0.02)	0.66 (0.04)	0.2 (0.02)	0.42 (0.03)	3.4 (0.3)	-1.3 (0.7)	-0.8 (0.9)	-0.7 (0.1)

Results for metrics from left to right: activity intensity, $M2_{ACC}$, $M30_{ACC}$, $M60_{ACC}$, PC1 and PC2 for DE50-MD and WT control dogs. Mean (standard deviation) displayed for each metric at each age between 3 and 18 months. Numbers varied at each age, see Table S1 for further details. Metrics counting discrete bouts have been rounded to the nearest integer, acceleration metrics (MX_{ACC}) to two decimal places and all other metrics to one decimal place. Symbols indicate group effect at all ages: * P<0.05, ** P<0.01, ***P<0.001. PC1 and PC2 refer to principal components 1 and 2.
